# Elevated serum OX40L is a biomarker for identifying corticosteroid resistance in pediatric asthmatic patients

**DOI:** 10.1186/s12890-019-0819-5

**Published:** 2019-03-19

**Authors:** Su-Li Ma, Lei Zhang

**Affiliations:** Department of Pediatrics, Pudong New District People’s Hospital, Shanghai University of Medicine & Health Sciences, No.490 South Huanchuan Road, Pudong New District, Shanghai, 201200 China

**Keywords:** Asthma, Inhaled corticosteroids (ICS), OX40 ligand (OX40L), Steroid resistance (SR), Biomarker

## Abstract

**Background:**

Corticosteroids are widely used to control asthma symptoms, but steroid resistance (SR) is a common adverse reaction. Therefore, it is important to accurately predict the corticosteroid response of asthmatic patients. This study aims to evaluate the serum OX40 ligand (OX40L) in pediatric asthmatic patients, and to investigated its correlations with clinical characteristics and corticosteroid response.

**Methods:**

A total of 192 pediatric asthmatic patients with inhaled corticosteroid (ICS) therapy and 130 healthy controls were selected. Clinical data were collected, and the serum levels of immunoglobulin (IgE), interleukin-6 (IL-6), thymic stromal lymphopoietin (TSLP), and OX40L were measured by enzyme-linked immunosorbent assay (ELISA). The level of serum OX40L was compared between the steroid-sensitive asthma (SSA) and steroid-resistant asthma (SRA) groups.

**Results:**

The serum OX40L level in asthmatic patients (713.5 ± 165.7 pg/mL) was significantly higher than that of the healthy controls (238.6 ± 27.8 pg/mL) (*P* < 0.001), and significantly higher in SRA group (791.2 ± 167.9 pg/mL) than in SSA group (655.6 ± 138.8 pg/mL) (P < 0.001). The serum OX40L level showed a significant positive correlation with serum IgE, blood percentages of eosinophils and neutrophils, serum IL-6 and TSLP, and showed a negative correlation with asthma control test (ACT) score and forced expiratory volume in first second (FEV1%). Receiver operating characteristics (ROC) curve was performed to obtain a cutoff value of serum OX40L as 780 pg/mL (sensitivity = 58.5%; specificity = 86.4%), which can identify SRA in asthmatic patients. Multivariate logistic regression analysis showed that elevated serum OX40L (≥780 pg/mL), as well as lymphocytes (%), ACT score, serum IL-6 and TSLP, were independent predictors of SRA (OX40L ≥ 780 pg/mL: odds ratio = 4.188; 95% CI = 1.800–9.746; *P* = 0.001). The serum OX40L level was decreased after ICS treatment in asthmatic patients, and the reduction in serum OX40L was significant higher in SSA group compared with SRA group.

**Conclusion:**

High serum OX40L can be used as a biomarker to identify asthmatic patients with corticosteroid resistance, and the change in OX40L level also reflects the response to ICS treatment. These results suggest an association of OX40L with the pathophysiology, inflammation, and clinical outcomes of asthma. New agents targeting OX40L can provide more precise and personalized therapy for asthma.

## Introduction

Asthma is the most common chronic inflammatory airway disease in children in the world and is characterized by reversible airflow obstruction. The prevalence of asthma in children is between 5 and 20%, and there are approximately 300 million people worldwide [1]. Asthma is a highly heterogeneous disease that develops in many clinical forms or phenotypes, has different pathogenic mechanisms, and is caused by a variety of risk factors, such as allergen exposure, viral infections, oxidative stress, and air pollution. Asthma is characterized by airway hyperresponsiveness (AHR), eosinophilic airway inflammation, elevated serum IgE levels and blood eosinophil counts, increased mucus production, and reversible airway obstruction and remodeling [2]. T helper 2 (Th2) cells initiate asthma and produce characteristic cytokines such as IL-4, IL-5 and IL-13. These cytokines induce IgE class switching from B cell (IL-4), eosinophil infiltration (IL-5) and goblet cell hyperplasia (IL-13) [3]. These mechanisms promote the maintenance of high IgE levels, infiltration of inflammatory cell tissue, eosinophilia, mucus release and smooth muscle contraction, resulting in severe allergic symptoms such as rhinitis and dermatitis. Therefore, assessment of airway inflammation is of great significance for improving the severity and preventing the recurrence of asthma. While direct airway sampling through bronchial biopsy or induced sputum is difficult to repeat in clinical settings, and systemic biomarkers of airway inflammation are desirable for evaluating severity of asthma.

Currently, inhaled corticosteroids (ICS) are widely recognized as first-line treatment of asthmatic patients, especially for pediatric patients [4]. However, a subset of asthmatics did not respond well to the ICS treatment and develop steroid resistance (SR), which has been considered as the most important reason for therapy failure [5]. Patients with steroid-resistant asthma (SRA) usually have persistent airway inflammation, prolonged high-dose ICS treatment, and unwanted side effects [6]. SR has become a challenging health problem due to the increased prevalence of asthma worldwide, thereby contributing to high cost asthma care. Therefore, it is of great importance to early identify SRA patients for providing effective personalized treatment and avoiding the associated side effects in asthmatic patients.

OX40 ligand (OX40L, CD252) and its receptor OX40 (CD134) are members of the tumor necrosis factor (TNF)/TNF receptor superfamily. OX40L is mainly expressed on B lymphocytes, dendritic cells, Langerhans cells and macrophage cells [7]. Increased expressions of OX40 and OX40L were found in the airway lamina propria of asthmatic patients [8], and promoted the polarization of naive T cells, thus produced large amount of Th2 cytokines, such as IL-4, IL-5, and IL-13 [9]. OX40 and OX40L protein levels in peripheral blood mononuclear cells (PBMCs) were significantly higher in asthmatic patients than those in controls group, and involved with enhanced proliferation capacity of T cells [10]. The elevated OX40L in airway lamina propria and PBMCs can release into circulation, as evidenced by higher serum OX40L in asthmatic children, especially those with severe and persistent asthma [11]. However, the association of serum OX40L with steroid sensitivity of pediatric asthmatic patients remains unclear.

In the present study, we hypothesized that, in primary pediatric patients with asthma, an upregulated serum OX40L will be associated with airway inflammation and steroid sensitivity. To test this hypothesis, serum concentrations of OX40L from steroid-resistant patients were compared with those of steroid-sensitive patients, and the relationships between OX40L levels and clinical characteristics were also examined.

## Methods

### Study population

A total of 192 children with asthma were included from July 2016 to December 2017, with age ranging from 4 to 10 years old. Patients are diagnosed by a physician according to the Global Initiative for Asthma (GINA) criteria [12]. None of the patients had experienced an upper respiratory infection or received systemic glucocorticoids within 8 weeks. In addition, 130 gender and age-matched healthy children were recruited as controls, with no history of atopy or respiratory disease. The clinical and laboratory data, and pulmonary function were collected before treatment to determine the serum total IgE, count and percentage of eosinophils, neutrophils, monocytes and lymphocytes, and FEV1% predicted. After baseline characterization, all 192 asthmatic patients received inhaled corticosteroid (ICS) therapy (budesonide, 400 μg/day) and β2 receptor agonist salbutamol (200 μg/day) for 7 days [13]. If the improvement rate of FEV1 was more than 10%, the patient was defined as steroid-sensitive asthma (SSA), or if there was less than 10% improvement in FEV1, the patient was defined as steroid-resistant asthma (SRA) [14]. Finally, all 192 asthmatic patients were divided into 110 SSA patients and 82 SRA patients. This study was approved by the Ethics Committee of the Pudong New District People’s Hospital and informed consent was obtained from the parents of patients.

### Serum samples collection

Venous blood (5 mL) was collected from all asthmatic patients before receiving ICS in the early morning and clotted at room temperature for 1 h. The sample was centrifuged at 2500 g for 15 min at 4 °C, and the supernatant was dispensed into 0.5 mL aliquots and stored at − 80 °C until measurement. All serum samples were processed according to standard protocols.

### ELISA assay

Serum concentrations of IL-6 (ab46027), TSLP (ab155444) and OX40L (ab213842) were determined by an ELISA kit (Abcam Co., Cambridge, UK), according to the manufacturer’s protocols and instructions. All samples were in triplicate and the average concentration for each patients was calculated.

### Statistical analysis

SPSS 19.0 statistical software (SPSS Inc., Chicago, IL, USA) was used to analyzed all data. Categorical variables were shown as frequency and percentage, and the comparison between different groups was performed using Chi-square test or Fisher’s exact test. Continuous variables were shown as mean and standard deviation, and differences between groups were determined using Student’s t-test and One-way analysis of variance (ANOVA) (normal distribution data) or Kruskal-Wallis test (nonnormal distribution data). Pearson correlation (normal distribution data) or Spearman’s rank correlation (nonnormal distribution data) was used to analyze the correlation between serum OX40L and other clinical characteristics of asthmatic patients. Receiver operating characteristics (ROC) curve was performed to determine the diagnostic value of serum OX40L for predicting SRA, with a cut-off point of 780 pg/mL. Multivariate Logistic regression analysis was used to determine independent variables for SRA, and was expressed as an odds ratio and a 95% confidence interval (CI). *P* < 0.05 was considered as a statistically significant difference.

## Results

### Clinical characteristics of asthma

**Fig. 1:**
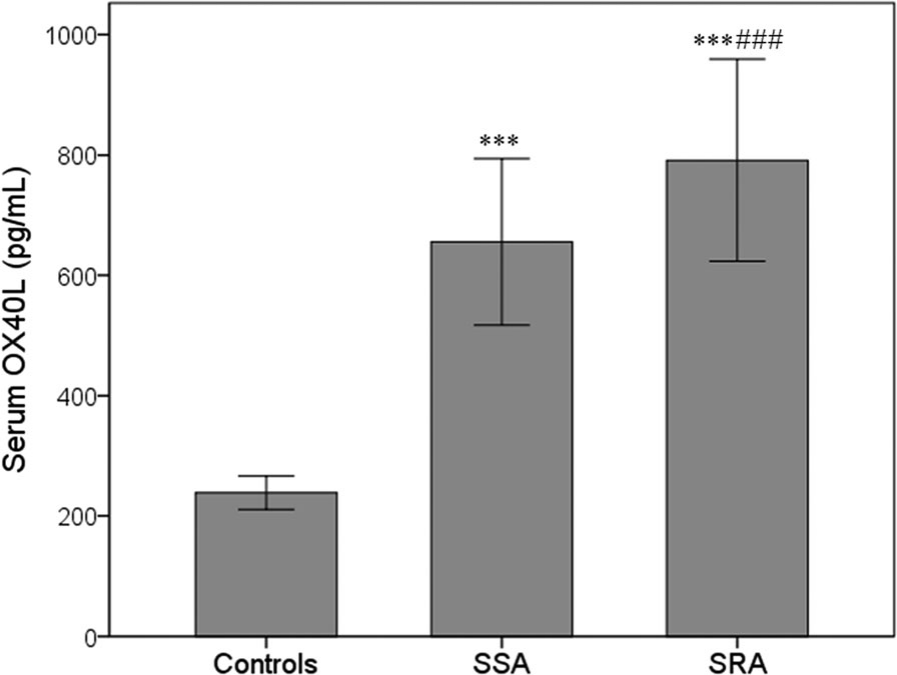
Comparison of serum OX40L level in control, SSA and SRA groups. The serum level of OX40L (mg/ml) are significantly increased in SRA group than in SSA and control groups. ***P < 0.001) vs control group; ### *P* < 0.001 vs SSA group. SSA: steroid-sensitive asthma; SRA: steroid-resistant asthma

A total of 192 pediatric asthmatic patients were enrolled in this study. The clinical characteristics and demographic profile of the patients are presented in Table 1. There were 110 patients with more than 10% improvement rate of FEV1, and were divided into SSA group, and 82 patients with less than 10% improvement rate of FEV1 were divided into SRA group. Compared to the SSA group, SRA group showed significantly higher serum IgE, count and percentage of blood eosinophils, neutrophils and lymphocytes, IL-6 and TSLP (All *P* < 0.05). Pulmonary function decreased significantly in SRA group compared with SSR group, as evidenced by lower FEV1%. The serum OX40L level in asthmatic patients (713.5 ± 165.7 pg/mL) was significantly higher than that of the healthy controls (238.6 ± 27.8 pg/mL) (*P* < 0.001). Furthermore, the serum OX40L level was 791.2 ± 167.9 pg/mL in SRA group, which was significantly higher compared with SSA group (655.6 ± 138.8 pg/mL) (Fig. 1).

**Table 1:** Serum OX40L and characteristics of subjects from SSA, SRA and control group

Parameter	Control group	SSA group	SRA group
Cases	130	110	82
Age (years)	6.78 ± 1.73	6.95 ± 1.72	7.01 ± 1.80
Gender (Male/Female)	67 (51.5%)	55 (50%)	42 (51.2%)
Atopic subjects	0	67 (60.9%)	48 (58.5%)
Serum IgE (IU/mL)	76.6 ± 14.4	281.5 ± 31.4*	292.2 ± 34.0*#
Eosinophil count (cells/mm^3^)	256.9 ± 50.7	597.1 ± 101.3*	630.5 ± 111.9*#
Eosinophils (%)	3.78 ± 0.75	7.66 ± 1.30*	8.08 ± 1.43*#
Neutrophil count (cells/mm^3^)	3749.8 ± 587.6	5073.9 ± 715.1*	5375.2 ± 743.8*#
Neutrophils (%)	55.14 ± 8.64	65.05 ± 9.17*	68.91 ± 9.54*#
Monocyte count (cells/mm^3^)	406.6 ± 91.7	529.3 ± 122.0	526.9 ± 136.5
Monocytes (%)	5.98 ± 1.35	6.79 ± 1.56	6.76 ± 1.75
Lymphocyte count (cells/mm^3^)	2626.3 ± 591.7	2370.0 ± 521.3*	2161.3 ± 423.9*#
Lymphocytes (%)	38.62 ± 8.70	30.39 ± 6.68*	27.71 ± 5.44*#
ACT score	25	16.82 ± 3.27*	13.98 ± 3.22*#
FEV1% predicted	99.2 ± 3.5	71.0 ± 9.3*	67.6 ± 9.5*#
IL-6 (pg/mL)	3.95 ± 0.72	6.94 ± 1.37*	8.24 ± 1.28*#
TSLP (pg/mL)	89.1 ± 12.1	223.2 ± 24.9*	244.5 ± 33.6*#
Serum OX40L (pg/mL)	238.6 ± 27.8	655.6 ± 138.8*	791.2 ± 167.9*#

Data are presented as means ± SD. Data in SSA and SRA groups were collected before inhaled corticosteroid (ICS) therapy. *P < 0.05 compared with control group; #*P* < 0.05 compared with SSA group. SSA: steroid-sensitive asthma; SRA: steroid-resistant asthma; IgE: immunoglobulin; ACT: asthma control test; FEV1: forced expiratory volume; IL-6: interleukin-6; TSLP: thymic stromal lymphopoietin; OX40L: OX40 ligand

### Association between serum OX40L with clinical parameters of asthmatic patents

To further investigate the clinical significance of serum OX40L in pediatric asthmatic patients, the Pearson correlation analysis was performed between serum OX40L and clinical characteristics of 192 asthmatic patients. Serum OX40L level was correlated positively to serum IgE, percentages of eosinophils and neutrophils, IL-6 and TSLP, and correlated negatively to ACT score and FEV1% (Table 2).

**Table 2:** The correlation between serum OX40L and clinical parameters

Clinical parameters	Serum OX40L	
	r	*P* value
Age (years)	−0.096	0.187
Serum IgE (IU/mL)	0.278	< 0.001
Eosinophils (%)	0.219	0.002
Neutrophils (%)	0.206	0.004
Monocytes (%)	0.017	0.816
Lymphocytes (%)	−0.132	0.067
ACT score	−0.235	0.001
FEV1% predicted	−0.374	< 0.001
IL-6 (pg/mL)	0.355	< 0.001
TSLP (pg/mL)	0.344	< 0.001

### High serum OX40L predicts corticosteroid resistance in asthmatic patients

**Fig. 2:**
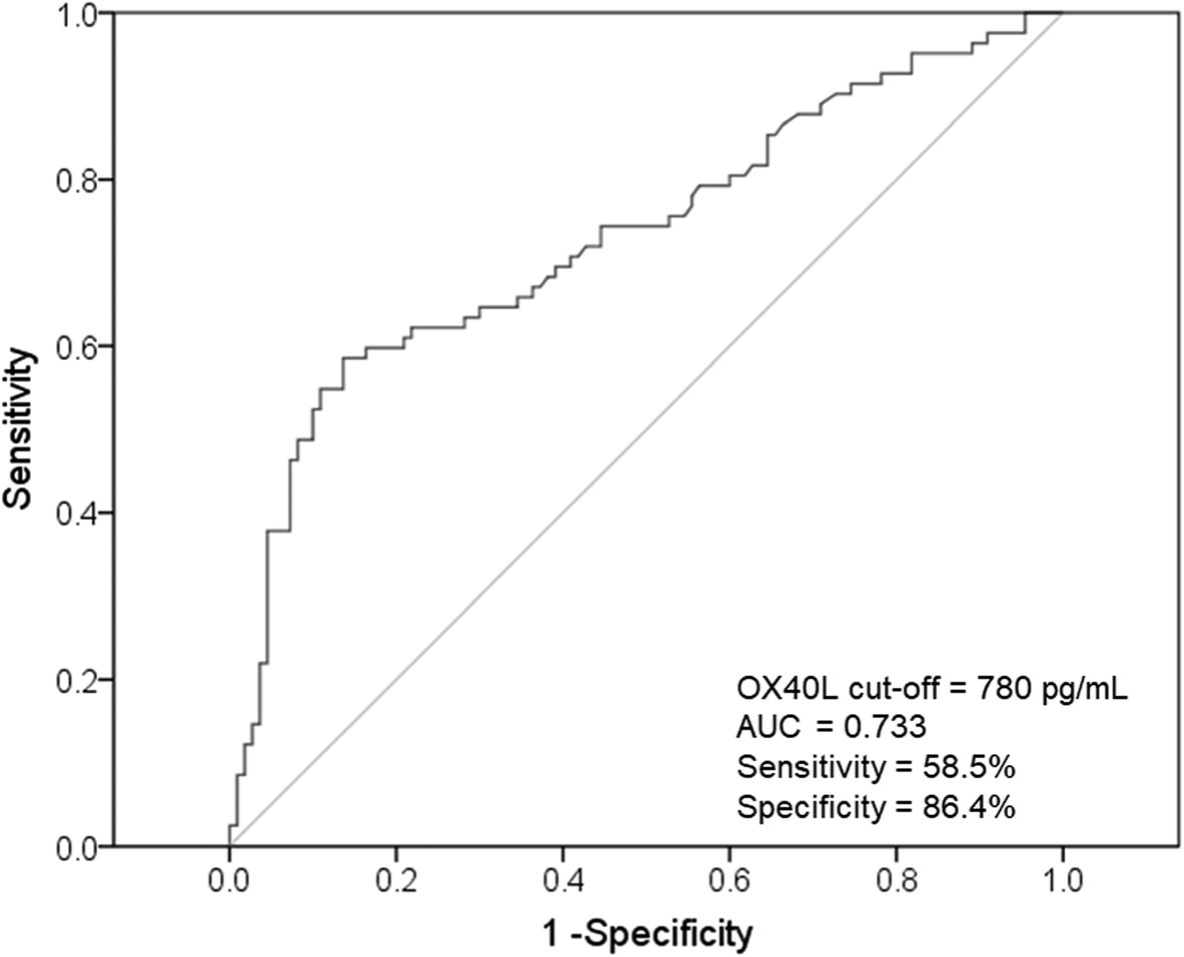
ROC curves of serum OX40L in predicting SRA in asthma patients. The AUC was 0.733. A cutoff value of VDBP is 780 pg/mL, which can discriminate SRA from SSA among asthma patients, with 58.5% sensitivity and 86.4% specificity. ROC: receiver operating characteristics; AUC: area under the curve

To evaluate the diagnostic value of the serum OX40L for prediction SRA, a receiver operating characteristic (ROC) curve analysis was performed. The area under curve (AUC) of OX40L was 0.733, and the cutoff for OX40L was selected as 780 pg/mL, with the sensitivity and specificity as 58.5 and 86.4%, respectively (Fig. 2). We further performed multivariate logistic regression analysis to evaluate whether serum OX40L is an independent predictor of corticosteroid response of asthmatic patients. The results showed that lymphocytes (%), ACT score, serum IL-6, TSLP and high OX40L were independent contributors of corticosteroid resistance (Table 3). Patients with higher serum OX40L (≥780 pg/mL) demonstrated significantly higher risk of SRA compared to patients with lower serum OX40L (< 780 pg/mL) (odds ratio = 4.188; 95% CI = 1.800–9.746; *P* = 0.001).

**Table 3:** Logistic multivariate regression predicts corticosteroid responsiveness

Clinical parameters	Odds Ratio	95% Confidence Interval	*P* value
Lymphocytes (%)	0.919	0.862–0.979	0.009
ACT score	0.759	0.671–0.858	<0.001
IL-6 (pg/mL)	1.889	1.385–2.576	<0.001
TSLP (pg/mL)	1.017	1.003–1.032	0.019
OX40L ≥ 780 pg/mL	4.188	1.800–9.746	0.001

Correlation was determined with the Spearman rank correlation coefficient

### Significant reduction of serum OX40L level in SSA patients

**Fig. 3:**
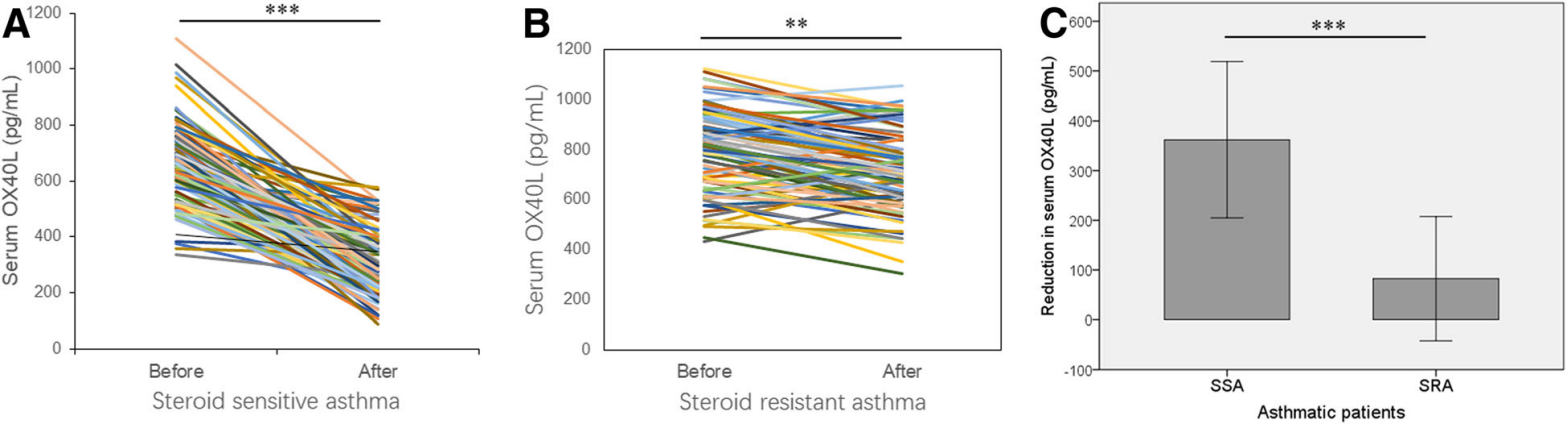
The change of serum OX40L level after ICS therapy in SSA (**a**) and SRA (**b**) asthmatic patients. **c** The reduction in serum OX40L after ICS treatment was compared between SSA group and SRA group. **P < 0.01; ***P < 0.001. SSA: steroid-sensitive asthma; SRA: steroid-resistant asthma

To evaluate the change of serum OX40L after ICS therapy, we compared the serum OX40L before and after treatment. The serum OX40L level decreased by 55.1% compared to the pretreatment period in patients of SSA group (655.6 ± 138.8 vs. 293.8 ± 113.3 pg/mL; *P* < 0.001) (Fig. 3a), while it reduced by 10.5% in SRA group (791.2 ± 167.9 pg/mL vs. 708.5 ± 159.2 pg/mL; *P* = 0.001) (Fig. 3b). The reduction in serum OX40L after ICS treatment was significant higher in SSA group compared with SRA group (*P* < 0.001) (Fig. 3c).

## Discussion

The present study shows that serum OX40L levels were significantly higher in pediatric patients with asthma than in healthy controls, and higher in steroid-resistant asthma (SRA) than in steroid-sensitive asthma (SSA). Among asthmatic patients, OX40L level positively correlated with airway inflammation, such as serum IgE, percentages of eosinophils and neutrophils, serum IL-6 and TSLP, and negatively correlated with asthma severity (ACT score) and pulmonary function (FEV1%). Serum OX40L is an independent risk predictor of SRA, with about 4 fold risk for patients with higher serum OX40L (≥780 pg/mL) compared with patients with lower serum OX40L (< 780 pg/mL). Inhaled corticosteroids (ICS) therapy decreased the serum OX40L levels, and the reduction of serum OX40L was more prominent in SSA than in SRA. These results suggest that serum OX40L could be a candidate biomarker for the identification of potential SRA among asthmatic patients, and patients with high OX40L might have more probability of corticosteroid resistance and require higher doses of corticosteroids for the initial ICS treatment. A rapid decline in the serum OX40L level reflect good response to corticosteroid treatment.

Our study shows the association of serum OX40L with airway inflammation, as evidenced by its positive correlation with serum IgE, IL-6, percentages of eosinophils and neutrophils. OX40/OX40L interactions regulate effector T cell function, such as CD4+ T cell response and survival [15], cytokine production [16] and the number of memory T cells [17]. Moreover, OX40/OX40L pathway also promoted allergic airway inflammation [18]. In ovalbumin (OVA)-induced asthma mice, OX40L protein treatment increased asthma severity, promoted inflammatory cytokine expression, T cell proliferation and eosinophil infiltration [19]. IL-6 is a proinflammatory cytokine and involves Th2/Th17-mediated airway inflammation and promotes airway hyperresponsiveness (AHR) [20]. This supports the fact that serum IL-6 is markedly higher in asthmatic patients compared with healthy controls [21], and higher in SRA compared with SSA in our results. The potential regulation of OX40L on eosinophilic infiltration was also confirmed in chronic rhinosinusitis, in which the number of OX40L-positive cells positively correlated with the number of eosinophils in nasal polyps [22]. Therefore, our results suggest that OX40L enhance airway inflammation in asthmatic patients.

In the present study, serum TSLP levels were significantly higher in SRA patients compared with SSA and controls, and demonstrated positive correlation with serum OX40L. TSLP is a cytokine produced from damaged epithelial cells that stimulates dendritic cells maturation and induces Th2-mediated inflammation [23]. OX40L is involved in TSLP-driven atopic inflammation and pathologic Th2 immune responses [24]. TSLP could activate dendritic cells and promote T follicular helper cells differentiation from naive CD4 T cells, which is depended on OX40L [25]. Thus, a TSLP-DC-OX40L pathway was formed and promoted airway inflammation and asthma pathogenesis by increasing inflammatory cytokines and decreasing CD4 + CD25 + Treg cells [26]. Therefore, OX40-OX40L constitute an important therapeutic target of asthma, and blockade of OX40L could disrupt TSLP-driven helper type 2 cells-related airway inflammation, thus diminish severe allergic symptoms [27].

Search for a sensitive biomarker to identify asthmatic patients with high risk to develop SRA is essential for improving asthma control. Currently, there are several serum biomarkers, including miRNA-21 and vitamin D-binding protein (VDBP), for predicting corticosteroid resistance of asthma [14, 28]. Our study adds OX40L as a new serum biomarker of corticosteroid resistance. OX40L is also an independent contributor of corticosteroid response of asthmatic patients, like lymphocytes (%), ACT score, serum IL-6 and TSLP. The role of OX40L in SRA was further confirmed by our results that the serum OX40L levels was decreased by corticosteroids therapy, and the reduction in serum OX40L was more prominent in SSA than in SRA. This indicates that serum OX40L is also a convenient indicator to monitor response to corticosteroid therapy. Moreover, inhibitors or blocking antibody of OX40L can be combined with corticosteroid in SRA patients with high serum OX40L.

The mechanisms underlying role of OX40L in corticosteroid response remain unclear. One recent report showed that TSLP might be an important contributor to corticosteroid resistance of asthma. Compared with healthy controls, TSLP in bronchoalveolar lavage (BAL) of asthmatic patients was increased, and BAL fluid TSLP levels showed markedly positive correlation with corticosteroid resistance of lung type 2 innate lymphoid cells (ILC2s) [29]. Given the fact that OX40L lies in the downstream of TSLP, whether OX40L or its receptor OX40 involve various mechanisms of corticosteroid resistance remains unclear, and deserves further investigation.

The limitations of the present study are as follows. Firstly, this clinical study is retrospective and relies on frozen serum and medical records. Prospective study is needed to examine the strength of serum OX40L on corticosteroid resistance. Secondly, this study mostly focused on the clinical significance of OX40L in patients with corticosteroid resistance and evaluated the prediction of ICS therapy response. The peripheral blood mononuclear cells (PBMCs) should be isolated from asthmatic patients and incubated in vitro with blocking antibody of OX40L, thereby to clearly elucidate the role of OX40L in corticosteroid resistance.

## Conclusions

Serum OX40L may be a useful noninvasive convenient indicator which could predict corticosteroid resistance in asthmatic patients. Serum OX40L correlated with airway inflammation indicators and is an independent predictor of SRA. So we speculate that OX40L might involve in the pathogenesis of corticosteroid resistance. Further study is needed to explore the role of OX40L in corticosteroid resistance and its regulation with TSLP in asthmatic animal model.

## Abbreviations

ACT: asthma control test
AHR: airway hyperresponsiveness
ANOVA: one-way analysis of variance
BAL: bronchoalveolar lavage
FEV1%: forced expiratory volume in first second
GINA: global initiative for asthma
ICS: inhaled corticosteroids
IgE: immunoglobulin
IL-6: interleukin-6
ILC2s: type 2 innate lymphoid cells
OVA: ovalbumin
OX40L: OX40 ligand
PBMCs: peripheral blood mononuclear cells
ROC: receiver operating characteristics
SR: steroid resistance
SRA: steroid-resistant asthma
SSA: steroid-sensitive asthma
Th2: T helper 2
TNF: tumor necrosis factor
TSLP: thymic stromal lymphopoietin
